# Size-based individualized tympanic membrane repair in elderly patients under otoendoscopy

**DOI:** 10.12669/pjms.42.2.13237

**Published:** 2026-02

**Authors:** Lei Yue, Juan Li, Yue Deng, Song Shi

**Affiliations:** 1 Lei Yue Department of Otolaryngology, Tongren Hospital, Shanghai Jiao Tong, University School of Medicine, Shanghai 200336, P.R. China; 2 Juan Li Department of Otolaryngology, Tongren Hospital, Shanghai Jiao Tong, University School of Medicine, Shanghai 200336, P.R. China; 3 Yue Deng Department of Otolaryngology, Tongren Hospital, Shanghai Jiao Tong, University School of Medicine, Shanghai 200336, P.R. China; 4 Song Shi Department of Otolaryngology, Tongren Hospital, Shanghai Jiao Tong, University School of Medicine, Shanghai 200336, P.R. China

**Keywords:** Cartilage tympanoplasty, Elderly patients, Myringoplasty, Otoendoscopy, Tympanic membrane perforation

## Abstract

**Objective:**

To explore the effectiveness and safety of different surgical methods based on perforation size selection for repairing tympanic membrane perforation (TMP) in elderly patients.

**Methodology:**

This retrospective analysis included clinical data of 156 elderly patients who underwent TMP repair surgery at Tongren Hospital Affiliated to Shanghai Jiao Tong University School of Medicine from January 2018 to December 2022. Patients were grouped based on the size of TMP: the Group-S (TMP size ≤ 2mm, n=48) underwent TMP repair using the butterfly technique; the Group-M (2mm < TMP size ≤ 4mm, n=62) underwent TMP repair by tragus cartilage tympanoplasty; the Group-L (TMP > 4mm, n=46) underwent repair using the tragus cartilage-perichondrium membrane composite. The surgical time, intraoperative blood loss, healing rate, air bone gap (ABG), and complications among the three groups of patients were compared.

**Results:**

The surgical time and intraoperative blood loss in Group-S were significantly lower than those in Group-M and Group-L (P<0.05). After a six months follow-up, the tympanic membrane healing rates ranged from 87.0% to 95.8% among the three groups, with no statistically significant difference observed (P>0.05). All groups demonstrated significant postoperative improvement in air-bone gap (ABG), while the magnitude of ABG reduction was greater in Group-S and Group-M than in Group-L (P<0.05). No significant difference in the incidence of postoperative complications was observed among the three groups (P>0.05).

**Conclusions:**

For elderly patients with TMP, different surgical procedures can be selected according to the size of the perforation, and can achieve a high tympanic membrane healing rate and satisfactory hearing improvement effect.

## INTRODUCTION

Tympanic membrane perforation (TMP) is a common otolaryngological disease in the elderly patients, caused by infection, trauma, or chronic otitis media, and is associated with a high incidence rate in this population.^1^,^2^ The self-healing rate of TMP in elderly patients is low due to the age-related decline in the tissue repair capacity, frequent combination of basic diseases (such as hypertension, diabetes, etc.), and repeated stimulation of chronic inflammation.^2^,^3^ If untreated, TMP can easily lead to progressive hearing loss, recurrent middle ear infections, and other complications, seriously affecting the quality of life.^3^

Tympanic membrane repair is the primary method for treating TMP, intending to seal the perforation, restore tympanic membrane integrity, improve hearing, and prevent middle ear infections.^4^ At present, commonly used endoscopic tympanic membrane repair techniques in clinical practice include internal implantation, external implantation, and dissection.^4^-^7^ The repair materials mainly include temporalis fascia, tragus cartilage membrane, tragus cartilage-chondral membrane complex, auricular cartilage, etc.^5^-^7^ However, due to significant differences in the etiology, size, and local tissue conditions of TMP among elderly patients, a single surgical procedure or material is often insufficient to meet the treatment needs of all patients.

Previous studies have shown that selecting individualized surgical procedures based on perforation size can improve surgical success rates.^8^ Butterfly repair is easy to perform and minimally invasive, and has therefore been widely applied in clinical practice.^9^ The use of a tragus cartilage–chondral membrane complex has also been reported to effectively seal TMP by taking advantage of the extensibility and biocompatibility of the fascia.^10^ However, large perforations remain challenging to repair because of their extensive perforation area and limited residual tympanic membrane tissue. In such cases, the tragus cartilage–perichondrium membrane composite may improve healing stability by providing stronger structural support and resistance to tension.^11^

However, although size-based individualized surgical strategies have been explored in previous studies, most investigations have focused on mixed-age populations or on comparisons of single surgical techniques or materials.^4^,^5^,^8^ Elderly patients represent a distinct subgroup, characterized by age-related decline in tissue healing capacity and a higher prevalence of comorbidities, which may influence surgical outcomes.^2^,^3^ To date, there is a lack of studies systematically evaluating the selection of surgical approaches based on perforation size specifically in elderly patients with TMP. Therefore, this study aimed to evaluate the feasibility, safety, and hearing outcomes of different surgical strategies selected according to perforation size in elderly TMP patients.

## METHODOLOGY

Clinical records of 156 elderly patients (156 ears) who underwent TMP repair surgery in Tongren Hospital Affiliated to Shanghai Jiao Tong University School of Medicine from January 2018 to December 2022 were retrospectively selected. Based on the size of the TMP (maximum diameter measured during surgery), patients were divided into three groups: Group-S (48 cases): perforation diameter ≤ 2mm; Group-M (62 cases): 2mm<perforation diameter ≤ 4mm; Group-L (46 cases): perforation diameter>4mm. Each group underwent TMP repair as follows: Group-S: butterfly technique; Group-M: tragus cartilage membrane implantation method; Group-L: tragus cartilage-perichondral composite graft.

TMP size was measured intraoperatively under otoendoscopic visualization. The maximum diameter of the perforation was determined using a sterile micro-ruler or a calibrated measuring probe routinely used in our department. A 0° otoendoscope was used as the primary viewing angle, and the endoscope was positioned as perpendicular to the tympanic membrane as possible to minimize parallax and magnification bias. All measurements were performed following a standardized protocol by experienced otologic surgeons from the same surgical team. In cases where the perforation margin was irregular or measurement uncertainty existed, the perforation size was confirmed by a second surgeon, and the final value was recorded by consensus.

### Ethical approval:

The Ethics Committee of Shanghai Tongren Hospital approved the study (Approval number K2024-015-01, Date: April 8, 2024).

### Inclusion criteria:

- Age ≥ 60 years old.
- Central perforation of the tense part of the tympanic membrane, confirmed by otoscopy and high-resolution computer tomography (HRCT) of the temporal bone, ruling out cholesteatoma and middle ear cholesteatoma.
- Perforation persists for ≥ three months without any self-healing trend.
- Pure tone audiometry shows conductive or mixed hearing loss, with an air-bone gap (ABG) of ≥ 10dB.
- Chronic medical conditions common in elderly patients (such as hypertension, diabetes mellitus) evaluated preoperatively and controlled within acceptable clinical ranges.
- Complete clinical and follow-up data.

### Exclusion criteria:

- Active or severe middle ear infection, cholesteatoma, or ossicular chain malformation.
- Severe inner ear dysfunction, defined as a bone conduction hearing threshold ≥ 60 dB.
- Severe systemic diseases (such as heart failure, hepatic or renal failure, or coagulation disorders) resulting in poor surgical tolerance.
- Narrowing, occlusion, or malformation of the external auditory canal.
- Previous history of middle ear surgery.

All surgical procedures were performed by a dedicated otologic surgical team at our institution. The surgeons involved were attending or senior otologic surgeons with comparable professional training backgrounds and extensive experience in otoendoscopic tympanic membrane repair. Throughout the study period, standardized surgical protocols and operative procedures were consistently applied across all cases to minimize surgeon-related variability.

### Group-S (Butterfly repair):

**Fig.1:**
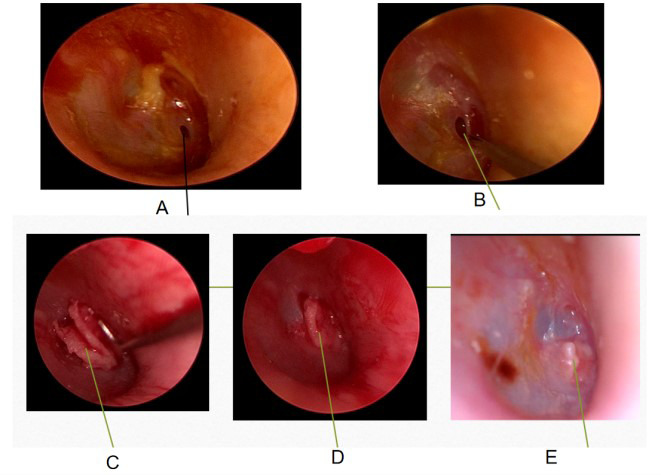
Female, 67 years old, with a perforation diameter of 1.8mm, underwent butterfly repair surgery. A. Schematic diagram of preoperative TMP; B. Creation of transplant wounds; C. Embedded butterfly-shaped cartilage plate; D. Good embedding of cartilage patch; E. After one month of postoperative follow-up, the tympanic membrane healed well.

Anesthesia method: Local anesthesia (1% lidocaine + 0.1% adrenaline for subcutaneous and tympanic membrane infiltration anesthesia in the external auditory canal), or general anesthesia for those who do not tolerate local anesthesia. Surgical steps:

- After disinfection of the external auditory canal, the TMP was exposed with an otoscope (0° or 30°), the epithelial tissue at the edge of the perforation was cleaned, and a fresh wound was formed.
- A crochet hook was used to remove the epithelium wrapped inside the eardrum and create a wound on the inner mucosal layer of the eardrum.
- The tragus cartilage was prepared, the excess soft tissue was removed, and the intact cartilage membranes were preserved on both sides. A blade was used to remove the edge of the cartilage and create a circular shape with a diameter 1.5-2 mm larger than the actual perforation.
- An 11^th^ blade was used to create deep grooves along the entire edge of the cartilage, forming a ‘butterfly shape’ on the side. The prepared graft was inserted into the perforated eardrum, with the “wings” located on both sides of the perforation;
- The area was fixed with gelatin sponge particles soaked in levofloxacin, dexamethasone, and growth factors under light pressure. (Fig.1)

### Group-M (tragus cartilage membrane implantation method):

**Fig.2:**
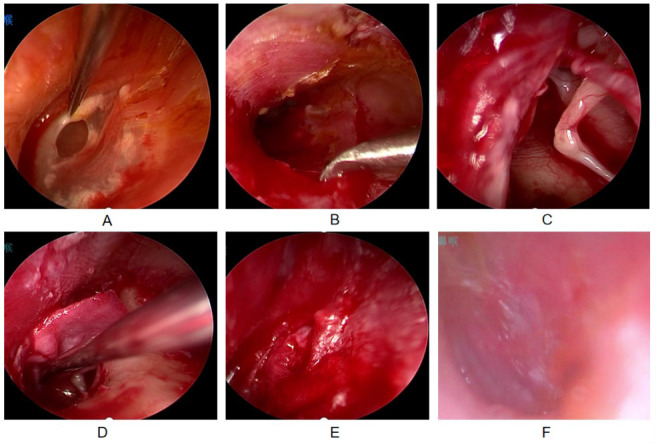
Male, 70 years old, with a perforation diameter of 3.5mm, underwent tragus cartilage membrane implantation via otoendoscopy. A. Making transplant beds; B. Lifting the skin flap; C. Placement of fascia; D. Resetting the skin flap; E. The built-in flap fits seamlessly without gaps. F. Two months after surgery, a follow-up examination showed good healing.

Anesthesia method was similar to the Group-S. Surgical steps:

- The TMP was exposed, the perforation edge and granulation tissue inside the tympanic cavity were cleaned to form a fresh wound.
- A crochet hook was used to remove the epithelium wrapped inside the eardrum and create a wound on the inner mucosal layer of the eardrum.
- 1:10000 adrenaline and infiltration anesthesia of the posterior upper wall of the external auditory canal were used, with a distance of 6-8mm from the tympanic ring. The skin of the external auditory canal was incised in the direction from 10:00 to 6:00. Care was taken to deeply reach the bone wall and open the external auditory canal flap forward, while paying attention to protecting the tympanic nerve. The drum ring was opened, and the middle ear cavity was entered to explore the range of motion of the ossicular chain.
- The cartilaginous membrane (approximately 1.0cm × 1.0cm) was removed from the parotid gland side of the ear, soaked in physiological saline, and flattened; the adipose tissue on the surface of the cartilaginous membrane was removed.
- The cartilage membrane was filled into the eardrum and flattened forward. The inner side of the cartilage membrane was filled with gelatin sponge soaked in levofloxacin and dexamethasone. Care was taken to ensure that the fascia was entirely placed on the inner edge of the perforation edge, more than 1mm beyond the perforation edge, and without tension.
- The residual tympanic membrane and external auditory canal skin flap were fixed with gelatin sponge particles soaked in levofloxacin, dexamethasone, and growth factors under light pressure. (Fig.2)

### Group-L (tragus cartilage-perichondral membrane composite):

**Fig.3:**
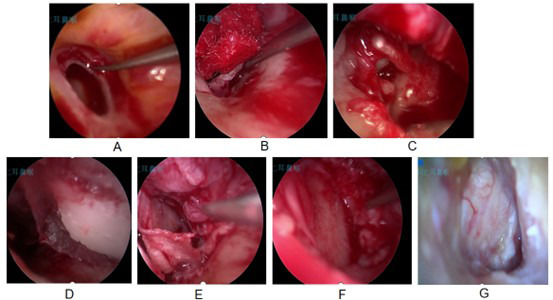
Male, 69 years old, with a perforation diameter of 5.8mm, underwent repair with tragus cartilage-perichondrium membrane graft; A. Making transplant beds; B. Opening the drum ring; C. Exposing the shaft of the hammer bone; D. Placement of a graft; E. The groove is embedded in the shaft of the hammer, and the cartilage is placed on the inner side. F. Resetting the skin flap and placing the perichondrium membrane on the outer side. G. Follow-up one month after surgery showed good healing.

Anesthesia method: General anesthesia (tracheal intubation was commonly used for general anesthesia in elderly patients to facilitate intraoperative monitoring). Surgical steps:

- After exposing the TMP, the perforation edge and granulation tissue inside the tympanic cavity were cleaned to form a fresh wound.
- The epithelium wrapped inside the eardrum was removed using a crochet hook, and a wound was created on the inner mucosal layer of the eardrum.
- 1:10000 adrenaline administration and infiltration anesthesia of the posterior upper wall of the external tract were performed. The skin of the external auditory canal was cut open 6-8mm from the drum ring from 10:00 to 6:00. The skin flap of the external auditory canal was opened forward to deeply reach the bone wall and protect the tympanic nerve. The tympanic membrane was opened and the middle ear cavity was entered. The tympanic membrane was then peeled off from the malleus handle from top to bottom to explore whether there is calcification in the anterior malleus ligament and investigate the range of motion of the ossicular chain.
- An 8-10mm of tragus cartilage with the unilateral cartilage membrane was taken. The tragus cartilage-perichondrium membrane graft was prepared, with cartilage size matching the size of the perforation. The cartilage membrane was 1-1.5mm larger than the cartilage, leaving a 0.5mm “gap” between the cartilage and the cartilage membrane. A 0.5mm “groove” in the middle of the cartilage was prepared.
- The tragus cartilage-perichondrium membrane graft was inserted into the middle ear cavity. The “groove” was inserted in the middle of the cartilage into the shaft of the malleus, the cartilage was placed on the inner side of the residual edge of the tympanic membrane, and the perichondrium membrane was placed on the outer side of the residual edge of the tympanic membrane.
- The external auditory canal flap was repositioned and fixed with gelatin sponge particles soaked in levofloxacin, dexamethasone, and growth factors under light pressure. (Fig.3)

### Postoperative management:

All patients underwent routine antibiotic treatment (cephalosporins or quinolones) for 3-5 days to prevent infection after surgery. Patients were instructed to keep the external auditory canal dry and avoid getting water, blowing the nose, and stimulating the external auditory canal. Follow-ups were done at one week, two weeks, one month, three months, and six months after surgery. The healing of the tympanic membrane, the condition of the external auditory canal, and the middle ear were monitored. Postoperative complications were assessed throughout the follow-up period (from one week to six months), and events were recorded based on findings at any follow-up visit, with the final complication rate summarized at the 6-month visit. Pure tone audiometry was performed at three and six months after surgery to evaluate the improvement of hearing.

### Therapeutic effect evaluation:

- Healing rate of tympanic membrane was evaluated by otoscopy. Healed: six months after surgery, the tympanic membrane is intact, and there is no perforation. Unhealed: all other cases;
- Hearing improvement: the preoperative and postoperative six months pure tone audiometry ABG were compared. ABG reduction value (preoperative ABG - postoperative ABG) was calculated;
- *Surgical indicators:* surgical time (from incision to closure), intraoperative blood loss. Surgical time was defined as the duration from the initiation of external auditory canal incision or operative manipulation to the completion of tympanic membrane repair and final packing of the external auditory canal. Intraoperative blood loss was primarily estimated based on the volume collected in the suction canister after subtracting irrigation fluids, and was supplemented by weighing blood-soaked gauze when applicable. All surgical indicators were recorded intraoperatively according to a standardized protocol.
- *Complications:* including middle ear infection, tympanic membrane reperforation, cholesteatoma formation, external auditory canal stenosis. Middle ear infection was defined as the occurrence of clinical symptoms (such as otorrhea and/or otalgia) accompanied by otoscopic/otoendoscopic evidence of middle ear inflammation during follow-up and requiring anti-infective treatment. Tympanic membrane reperforation was defined as the reappearance of a full-thickness tympanic membrane perforation after initial postoperative closure. External auditory canal stenosis was defined as clinically significant narrowing observed during follow-up that interfered with examination or required intervention. All complications were managed according to standard otologic protocols, and outcomes were documented in the medical records.

### Statistical analysis:

All analyses were done using SPSS version 27.0 (IBM Corp, Armonk, NY, USA). Continuous variables were reported as mean and standard deviation (SD). The one-way analysis of variance (ANOVA) was used to evaluate the statistical significance of the differences in continuous variables between the three groups, and conduct pairwise comparisons using the LSD method afterwards. A p-value less than 0.05 was considered statistically significant; all reported p-values were two-sided.

## RESULTS

This study included a total of 156 patients (68 males and 88 females), aged 60-82 years, with an average age of 67.5±5.3 years. The course of illness ranged from 0.5 to 15 years, with an average of 4.2±2.1 years. Causes of perforation included chronic otitis media (n=102), trauma (n=36), and other causes, such as iatrogenic injury (n=18). There was no statistically significant difference in general information between the three groups of patients (P>0.05) (Table-I).

**Table-I:** Comparison of General Information of Three Groups of Patients.

Variables	Group-S (n=48)	Group-M (n=62)	Group-L (n=46)	χ^2^/t	P
Gender (Male/Female), n(%)	20/28	27/35	21/25	0.324	0.85
Age (years), Mean±SD	66.8±4.9	67.6±5.5	68.1±5.7	0.587	0.557
Disease duration (years), Mean±SD	3.9±1.8	4.3±2.2	4.5±2.3	0.872	0.419
Pathogeny, n(%)				1.563	0.457
Chronic otitis media	32(66.7)	41(66.1)	29(63.0)		
Trauma	12(25.0)	15(24.2)	9(19.6)		
Others	4(8.3)	6(9.7)	8(17.4)		

The surgical time and intraoperative blood loss in the Group-S were significantly lower than those in the Group-M and the Group-L (P<0.05). The surgical time and intraoperative blood loss in the group with medium perforation were significantly lower than those in the group with large perforation (P<0.05) (Table-II).

**Table-II:** Comparison of surgical indicators among three groups of patients, Mean ± SD.

Group	n	Surgical time(min)	Intraoperative blood loss(mL)
Group-S	48	35.2±8.6	0.51±0.23
Group-M	62	58.5±12.3	1.26±0.45
Group-L	46	82.3±15.7	2.58±0.67
*F*		102.365	9.8742
*P*		<0.001	<0.001

As shown at the six months follow-up, healed TMP was reported in 46 patients in Group-S (a healing rate of 95.8%), 56 patients in Group-M (a healing rate of 90.3%), and 40 patients in Group-L (a healing rate of 87.0%). There was no statistically significant difference in the healing rate among the three groups (χ ^2^=2.154, P=0.341) (Table-III).

**Table-III:** Comparison of tympanic membrane healing rates among three groups of patients, n (%).

Group	n	Healed	Unhealed	Healed rate(%)
Group-S	48	46	2	95.8
Group-M	62	56	6	90.3
Group-L	46	40	6	87.0
*χ^2^*				2.154
*P*				0.341

There was no statistically significant difference in ABG among the three groups of patients before surgery (P>0.05). Six months after surgery, all three groups showed a significant reduction in ABG compared to preoperative levels (P<0.05). While there was no statistically significant difference (P>0.05) in the postoperative ABG reduction values between the Group-S and the Group-M, the postoperative ABG reduction value of the Group-L was significantly smaller than that of Group-S and Group-M (P<0.05) (Table-IV). As shown in Table-V, there was no statistically significant difference in the incidence of complications among the three groups (*χ^2^*=1.872, P=0.392) (Table-V). All complications were relieved after symptomatic treatment (anti-infection, local dressing change, etc.), and no serious adverse consequences occurred.

**Table-IV:** Comparison of ABG before and after surgery among three groups of patients (Mean ± SD, dB).

Group	n	Preoperative ABG	ABG at six months after surgery	ABG reduction value
Group-S	48	25.6±4.8	7.0±3.1*	18.6±5.2
Group-M	62	26.3±5.1	8.5±3.5*	17.8±4.9
Group-L	46	27.1±5.3	12.9±4.2*^ab^	14.2±4.3^ab^
F		0.632	28.754	15.327
P		0.532	<0.001	<0.001

*Note:* Compared with preoperative results in the same group,

* *P*<0.05; Compared with the Group-S, ^a^*P*<0.05; Compared with Group-M, ^b^*P*<0.05.

**Table-V:** Comparison of the incidence of complications among three groups, n (%).

Complications	Group-S (n=48)	Group-M (n=62)	Group-L (n=46)	χ^2^	P
Middle ear infection	1 (2.1)	2 (3.2)	1 (2.2)		
Recurrence of TMP	0 (0)	2 (3.2)	2 (4.3)		
Stricture of external auditory meatus	0 (0)	0 (0)	1 (2.2)		
Total incidence rate	1 (2.1)	4 (6.4)	4 (8.7)	1.872	0.392

## DISCUSSION

To our knowledge, this is the first comparison of different surgical methods, selected based on perforation size for repairing TMP in elderly patients. This study divided elderly patients into three groups based on the size of the perforation. Butterfly repair was used for small perforations, the tragus cartilage membrane implantation method was for medium perforations, and the tragus cartilage-perichondrium membrane graft was used for large perforations. The results showed that all three groups achieved high tympanic membrane healing rates (87.0%~95.8%) and satisfactory hearing improvement effects. This confirms the effectiveness of individualized surgical approach selection.

Small tympanic membrane perforations (diameter ≤ 2 mm) require limited mechanical support from repair materials due to their small perforation size, making butterfly repair an ideal choice.^12^,^13^ Butterfly repair utilizes the relatively humid environment inside the tympanic cavity to promote the healing of materials and the residual edge of the tympanic membrane, and has the advantages of easy operation, minimal trauma, and short surgical time.^13^,^14^ In this study, the surgical time and intraoperative bleeding in the Group-S were significantly lower than those of the other two groups, and the healing rate was as high as 95.8%. This is consistent with literature reports.^14^ Butterfly repair relies on the preparation of the “butterfly-shaped” repair material, which should be slightly larger than the perforation range of the tympanic membrane by 1.5-2 mm to ensure a tight fit between the repair material and the residual edge of the tympanic membrane.^12^-^14^

As elderly patients may have thickening and calcification of eardrums, it is necessary to thoroughly clean the epithelial tissue at the edge of the perforation to form a fresh wound and promote the growth of fibrous tissue.^15^ When making a transplant bed, it is essential to remove the epithelium that has inverted into the inner side of the tympanic membrane to avoid the formation of iatrogenic cholesteatoma. At the same time, it is necessary to create a mucosal surface transplant wound to increase the healing rate of the tympanic membrane.^15^,^16^ In this study, only one case of middle ear infection occurred in the Group-S, which healed after anti-infection treatment, indicating that butterfly repair has a higher safety in small perforations.

Mid-size perforation (2mm<diameter ≤ 4mm) requires consideration of the ductility and healing stability of the repair material. When repairing the tympanic membrane under a microscope, the temporal fascia is commonly used as a material for making a posterior incision in the ear.^17^ The temporal fascia has the characteristics of convenient sampling, good biocompatibility, and abundant blood supply. Its thickness is moderate (about 0.1-0.2mm), suitable for implantation between the epithelial and fibrous layers of the tympanic membrane.^17^,^18^ But in the ear, it is a one-handed operation, and the texture of the temporal fascia is relatively soft, making it difficult to flatten.^18^ However, the texture of the cartilage membrane on the parotid side of the tragus is relatively rigid, making it easy to apply fascia with one hand.^19^

In this study, patients in Group-M were treated with parotid gland–side tragus cartilage membrane implantation, achieving a healing rate of 90.3% and an ABG reduction value of 17.8 ± 4.9 dB, which was not significantly different from the Group-S. This indicates that tragus cartilage membrane implantation can effectively repair perforations. Several technical points of tragus cartilage membrane implantation need to be considered:

- The tragus cartilage membrane implantation trimming should be smooth to avoid wrinkles.
- It is important to ensure that the implant covers the perforation without tension during implantation, with the edge extending more than 1mm beyond the perforation to ensure healing strength.
- If there is fluid accumulation or granulation tissue in the tympanic cavity, it should be thoroughly cleaned to avoid affecting the survival of the fascia.^17^-^19^ Elderly patients may have atrophy of the tympanic mucosa, and excessive scratching should be avoided during surgery to prevent damage to the blood vessels of the tympanic mucosa and affect the blood supply.^20^ All six unhealed patients in the perforation group experienced fascial displacement or infection, indicating that postoperative care (such as avoiding blowing the nose and keeping the ear canal dry) is crucial for the healing of central perforation.

Large perforations (diameter>4mm) are significantly more difficult to repair due to the lack of residual tympanic membrane and high tension. Elderly patients have poor tissue elasticity, and traditional fascial repair is prone to reperforation due to excessive tension.^20^,^21^ The tragus cartilage has good support, tension resistance, and anti-infection ability, making it an ideal material for repairing large perforations.^22^ The Group-L in this study underwent repair using the tragus cartilage-perichondrium membrane graft, with a healing rate of 87.0%. While the healing rate was lower than that of Group-S and Group-M, it is still relatively high, with controllable complications. There are several obvious advantages of the tragus cartilage-perichondrium membrane graft. The rigid structure of cartilage can effectively resist pressure inside the tympanic cavity, and the “sandwich” method that uses cartilage and cartilage membrane to “sandwich” the residual edge of the tympanic membrane inside and outside prevents sinking that is caused by simple cartilage embedding. Additionally, during the operation, the tympanic membrane is peeled off from the malleus bone handle, making it easy to explore the activity of the ossicular chain and clear the lesions in the anterior and posterior tympanic canals. This method allows for easy exposure of the anterior malleus ligament, cleaning up calcified lesions, and improving postoperative hearing. Furthermore, the cartilage membrane is rich in fibroblasts, which can promote healing of the residual edge of the tympanic membrane. The tragus cartilage is easy to obtain and has a similar shape to the tympanic membrane. Therefore, the postoperative recovery of tympanic membrane function is closer to the physiological state.^23^,^24^

From a biomechanical perspective, the physical properties of graft materials, such as thickness and stiffness, may influence tympanic membrane vibration patterns after reconstruction.^16^–^18^,^22^ These material-related factors can affect sound energy transmission through the reconstructed membrane and ossicular chain, thereby partially explaining differences in postoperative hearing outcomes.^16^,^17^,^22^

The ABG reduction value (14.2 ± 4.3 dB) of the Group-L was significantly lower than that of the other two groups. This may be related to the fact that large perforations are often combined with restricted or hardened ossicular chain activity.^25^ Alternatively, it may be related to the increased energy consumption of sound waves during transmission caused by thicker cartilage compared to fascia.^26^ Therefore, combining preoperative HRCT assessment of the ossicular chain with ossicular chain reconstruction surgery may further improve hearing.^27^

Six months after surgery was selected as the primary follow-up time point for hearing evaluation in the present study. In tympanic membrane repair, this time point is commonly used to assess initial graft integration and stabilization of hearing outcomes, as tympanic membrane healing and postoperative air-bone gap improvement generally reach a relatively stable stage within this period.^5^,^6^,^8^ Using a 6-month endpoint also allows comparison with a substantial proportion of previously published studies on tympanic membrane repair.^5^,^14^

During the repair of TMP in elderly patients, special attention should be paid to:

- *Preoperative evaluation*. A comprehensive assessment of heart, lung, liver, kidney function, blood glucose, and blood pressure control is imperative for the reduction of surgical risks.
- *Anesthesia selection*. Local anesthesia can be used for small and medium perforations, while general anesthesia is recommended for large perforations to facilitate fine operation and vital sign monitoring during surgery.
- *Intraoperative procedure*. In elderly patients with fragile skin on the eardrum and external auditory canal, excessive pulling should be avoided to reduce tissue damage.
- *Material selection*. Priority should be given to materials with abundant blood supply and strong anti-infection ability (such as cartilage with perichondrium membrane) to adapt to the poor tissue healing ability of elderly patients.
- *Postoperative management*. Elderly patients will benefit from the extended follow-up time, anti-infective treatment, monitoring hearing changes, and promptly handling complications.

### Strengths of the study:

The strengths of the present study merit emphasis. This study specifically focused on elderly patients with tympanic membrane perforation, a population that presents distinct anatomical, physiological, and perioperative characteristics and remains relatively underexplored in the existing literature. In addition, with a relatively large sample size for a single-center study, our findings provide robust real-world evidence supporting the feasibility and safety of a size-based individualized surgical strategy under otoendoscopy. By integrating perforation size with endoscopic surgical decision-making, this study offers a practical framework that may assist clinicians in optimizing procedure selection for elderly patients.

### Limitations:

First, this was a retrospective, single-center analysis with a relatively limited sample size and a follow-up duration of six months; therefore, late events such as delayed healing, reperforation, or changes in long-term hearing stability beyond 12 months could not be fully assessed, particularly in elderly patients. Second, TMP etiologies were not analyzed in subgroups, and potential etiology-related differences in healing and hearing outcomes were not explored. Third, only the repair materials and techniques used in our routine practice were evaluated, and other commonly used graft materials (e.g., temporalis fascia or synthetic repair membranes) were not included for comparison. Fourth, although the surgeries were performed according to standardized protocols by a dedicated otologic surgical team, surgeon-dependent variability and differences in case complexity may still have influenced operative indicators and outcomes. Future prospective, multicenter studies with larger sample sizes and longer follow-up are warranted to validate and refine the proposed size-based surgical strategy in elderly patients.

## CONCLUSIONS

For elderly patients with tympanic membrane perforation (TMP), personalized surgical methods based on perforation size have significant advantages. Butterfly repair may be used for small perforations, with minimal trauma and fast recovery. The tragus cartilage membrane implantation can achieve both healing rate and hearing improvement in cases of medium-sized perforation. Patients with large perforations may benefit from the tragus cartilage-perichondrium membrane grafting, which can improve the stability of healing through the strong support of cartilage. All three surgical procedures demonstrated high tympanic membrane healing rates, satisfactory clinical efficacy, and acceptable safety under the conditions of this retrospective single-center study. Further prospective, multicenter studies with longer follow-up are warranted to validate their long-term effectiveness and broader clinical applicability.

### Recommendations:

Future research should further expand upon these findings. Prospective and multicenter studies are encouraged to validate the proposed size-based surgical strategy in broader clinical settings. Longer follow-up periods will be valuable for evaluating sustained hearing outcomes and long-term tympanic membrane integrity in elderly patients. In addition, future investigations may incorporate elderly-specific factors, such as comorbidity burden, Eustachian tube function, and functional quality-of-life outcomes, to further refine individualized surgical decision-making.

### Authors’ contributions:

**LY and SS:** Study design, literature search and manuscript writing.

**LY, JL and YD:** Data collection, data analysis and interpretation. Critical Review.

**SS:** Manuscript revision and validation and is responsible for the integrity of the study.

All authors have read and approved the final manuscript.
